# Numerical Study of Laminar Flow and Convective Heat Transfer Utilizing Nanofluids in Equilateral Triangular Ducts with Constant Heat Flux

**DOI:** 10.3390/ma9070576

**Published:** 2016-07-15

**Authors:** Hsien-Hung Ting, Shuhn-Shyurng Hou

**Affiliations:** Department of Mechanical Engineering, Kun Shan University, Tainan 71070, Taiwan; e056ting@gmail.com

**Keywords:** nanofluids, heat transfer enhancement, Nusselt number, Peclet number

## Abstract

This study numerically investigates heat transfer augmentation using water-based Al_2_O_3_ and CuO nanofluids flowing in a triangular cross-sectional duct under constant heat flux in laminar flow conditions. The Al_2_O_3_/water nanofluids with different volume fractions (0.1%, 0.5%, 1%, 1.5%, and 2%) and CuO/water nanofluids with various volume fractions (0.05%, 0.16%, 0.36%, 0.5%, and 0.8%) are employed, and Reynolds numbers in the range of 700 to 1900 in a laminar flow are considered. The heat transfer rate becomes more remarkable when employing nanofluids. As compared with pure water, at a Peclet number of 7000, a 35% enhancement in the convective heat transfer coefficient, is obtained for an Al_2_O_3_/water nanofluid with 2% particle volume fraction; at the same Peclet number, a 41% enhancement in the convective heat transfer coefficient is achieved for a CuO/water nanofluid with 0.8% particle volume concentration. Heat transfer enhancement increases with increases in particle volume concentration and Peclet number. Moreover, the numerical results are found to be in good agreement with published experimental data.

## 1. Introduction

The performance of convective heat transfer devices for single phase flows with relatively low thermal characteristics of heat transfer fluids (such as water, engine oil, and ethylene glycol) can be greatly improved by many augmentation techniques. Nanofluid is a new class of heat transfer fluids. The use of nanofluids for heat transfer enhancement is one of the passive heat transfer techniques in many heat transfer applications. Solids have greater thermal conductivity than liquids. Thus, dispersing nanoparticles, generally a metal or metal oxide, in fluids can greatly improve the thermal conductivity of liquids and, in turn, can help to improve their thermal characteristics (Nasiri et al. [1]).

Duct geometry is one of the essential factors influencing the pressure drop and heat transfer under laminar and turbulent flow conditions [2]. The literature indicates that studies have mainly focused on the convective heat transfer characteristics of fluids in a circular tube. The main reason is the heat transfer rate of these non-circular ducts is lower when compared to circular tubes. However, in fact, the pressure drop of a non-circular (such as triangular and square) duct is much less than that of a circular tube. Due to size, volume, and pressure drop limitations, increased effort is being put into the use of non-circular flow passage geometries for heat transfer applications in industries such as compact heat exchangers, aerospace, nuclear, biomedical engineering, and electronics [3,4,5,6]. In particular, the triangular ducts were utilized because they are more easily produced and have higher compaction, as well as less pressure drop when compared to other ducts. These reasons cause heat transfer enhancement of noncircular ducts, especially triangular ducts, to become a very important issue for their applications in different industries [3,4,5,6]. Heat transfer augmentation using nanofluids in a triangular duct provides this opportunity to tackle the problem of a lower heat transfer rate and to significantly improve the heat transfer performance.

Heris et al. [3] performed an experimental study to determine the pressure drop and heat transfer characteristics of Al_2_O_3_/water and CuO/water nanofluids in a triangular duct under constant heat flux where the flow was laminar. Their results showed that, at the same values of nanoparticle volume fraction and Reynolds number, using Al_2_O_3_ nanoparticles is more beneficial than CuO nanoparticles.

Heris et al. [4] experimentally investigated the heat transfer of an equilateral triangular duct by employing an Al_2_O_3_/water nanofluid under laminar flow and constant heat flux conditions. They estimated Nusselt numbers for different nanoparticle concentrations at various Peclet numbers. It was found that the heat transfer coefficient of Al_2_O_3_/water nanofluid is higher than that of distilled water. Additionally, they pointed out that the heat transfer enhancement increases with increases in the nanoparticle volume concentration and Peclet number.

Heris et al. [6] numerically investigated laminar forced convective heat transfer of Al_2_O_3_/water nanofluid in a triangular duct under constant wall temperature condition. It was found that the nanofluid Nusselt number increases with increasing particle concentration and decreasing particle diameter, and that the heat transfer enhancement becomes better at a higher Reynolds number in laminar flow using nanofluids.

Akbarzadeh et al. [7] performed a sensitivity analysis on the nanofluid heat transfer in a wavy channel. Their results indicated that at a fixed Reynolds number (Re = 600) and aspect ratio (0.1), the increment in the Nusselt number and the pressure drop up to 24% and 25%, respectively, with an increase in the solid volume fraction of nanoparticle.

In addition to Newtonian nanofluids, convective heat transfer enhancement using non-Newtonian nanofluids has attracted a lot of interest from numerous researchers in recent years. Some investigations on the topic of non-Newtonian fluids were reported [8,9,10].

Electrohydrodynamic (EHD) natural convection of a nanofluid in the presence of an electric field has been investigated recently [11,12]. Sheikholeslami and Ellahi [11] studied Fe_3_O_4_-ethylene glycol nanofluid hydrothermal treatment in a lid-driven cavity with a sinusoidal upper wall subjected to a non-uniform electric field. They found that heat transfer was enhanced with the increasing strength of the filed (due to existence of thermal plumes) and Reynolds number (because of the decrease in the thermal boundary layer thickness). Furthermore, the electric field effect on heat transfer became stronger at a low Reynolds number. In a subsequent study, Sheikholeslami and Chamkha [12] examined heat transfer characteristics of electrohydrodynamic free convection of a Fe_3_O_4_-ethylene glycol nanofluid in a semi-annulus enclosure with a sinusoidal wall. It was found that heat transfer enhancement increased by increasing the voltage supplied and Rayleigh number. The effect of the electric field on heat transfer is more marked at low Rayleigh numbers because of the predomination of the conduction mechanism.

Moreover, magnetohydrodynamic (MHD) flows have been widely investigated. Zeeshan et al. [13] utilized a body math mathematical analysis to investigate the effects of magnetic dipole and thermal radiation on the flow of viscous ferromagnetic fluid past a stretching sheet. Rashidi et al. [14] employed the Darcy-Brinkman-Forchheimer model to investigate the two-dimensional fluid flow with heat transfer around an obstacle embedded in a porous medium under the influence of a stream-wise transverse magnetic field. It was shown that the effects of a transverse magnetic field on flow behavior and the heat transfer mechanism are more than that of the stream-wise magnetic field. Rahman et al. [15] examined the combined effects of nanoparticles and slip for the blood flow of Jeffrey fluid in a tapered artery with stenosis. Sheikholeslami and Ellahi [16] used the Lattice Boltzmann method to simulate three-dimensional nanofluid flow and heat transfer in the presence of a magnetic field inside an enclosure (cubic cavity). They found that the average Nusselt number increased with an increase in Rayleigh number and with a decrease in Hartmann number. Ellahi et al. [17] investigated the natural convection boundary layer flow along a vertical cone with variable wall temperature in the presence of magnetohydrodynamics by means of the salt-water solution-based nanofluids with single and multi-wall carbon nanotubes. Additionally, Ellahi et al. [18] studied the natural convection boundary layer flow of nanofluids on entropy generation along an inverted cone. Their results indicated that the Nusselt number and skin friction decreased by increasing the magnetic and porosity parameters, while they increased with an increase in nanoparticle volume fraction and viscous dissipation. Sheikholeslami et al. [19] analyzed thermal radiation on a magneto- hydrodynamic nanofluid by means of a two phase model. They reported that the Nusselt number increased with thermal radiation and the Reynolds number. Kandelousi and Ellahi [20] investigated the influence of a magnetic field on Fe_3_O_4_-plasma nanofluid flow in a vessel as a targeted drug delivery system. It was found that the presence of a magnetic field greatly influenced the flow field, and that the skin friction coefficient decreased by increasing the Reynolds number and magnetic number. Akbar et al. [21] examined interaction of carbon nanotubes for the peristaltic flow with the induced magnetic field, and found that pressure gradient increased with increase in nanoparticle volume fraction, magnetic Reynolds number, and Strommer’s number, while the pressure gradient decreased with an increase in heat generation and heat flux. Ellahi et al. [22] studied the effects of aggregations on two-dimensional heat transfer mixed convection flow of an engine oil-based nanofluid with Fe_3_O_4_ nanoparticles over a vertical stretching permeable sheet. Their results showed that, with increasing the nanoparticle volume fraction, the velocity of the nanofluid decreased, while the temperature of the nanofluid increased.

In recent years, much attention has been focused on the heat transfer characteristics of nanofluids in circular tubes. Relatively few experimental and theoretical studies have been conducted on convective heat transfer of nanofluids using non-circular ducts [23], particularly a triangular duct, under a constant wall heat flux condition. In this study, we aim to numerically investigate the characteristics of convective heat transfer of water-based Al_2_O_3_ and CuO nanofluids flowing in a triangular duct with a constant heat flux under laminar flow conditions. The nanoparticle size of Al_2_O_3_/water is 20 nm and five particle concentrations (ϕ) of 0.1, 0.5, 1, 1.5, as well as 2 vol. % are considered. The water-based Al_2_O_3_ nanofluids flow through a triangular cross-sectional duct with a hydraulic diameter of 4.2 mm. The nanoparticle size of CuO/water nanofluid is 50 nm and five particle concentrations of 0.05, 0.16, 0.36, 0.5, as well as 0.8 vol. % are considered. The water-based CuO nanofluids flow through a triangular cross-sectional duct with a hydraulic diameter of 3.5 mm. Furthermore, the average Nusselt number and convective heat transfer coefficient obtained from the present numerical study are compared with those of Heris et al.’s experimental studies [4,5].

## 2. Mathematical Modeling

### 2.1. Assumptions and Governing Equations

The single-phase approach for nanofluids [24,25] is adopted in this numerical study. The base fluid and nanoparticles are assumed to be perfectly mixed and, thus, can be treated as a homogeneous mixture. The flow is laminar and steady-state. Moreover, the fluid phase and solid particles are assumed to be in thermal equilibrium and move with the same local velocity considering the ultra-fine and low volume fraction of the solid particles. The thermophysical properties of the base fluid (water) and the solid nanoparticles (γ-Al_2_O_3_ and CuO) used in the present study are specified in Table 1 (Heris et al. [4,5]).

The following nonlinear governing equations represent the mathematical formulation of the single-phase model, which include conservation of mass, momentum, and energy for the nanofluid flow inside the triangular cross-sectional duct.

Conservation of mass:

(1) $$div\;(\rho_{nf}\vec{V})=0$$

Conservation of momentum:

(2) $$div\;(\rho_{nf}\vec{V}\vec{V})=-\nabla P+\mu_{nf}\nabla^{2}\vec{V}$$

Conservation of energy:

(3) $$div\;(\rho_{nf}\vec{V}C_{pnf}T)=div\;(k_{nf}\nabla T)$$

where $\vec{V}$, *P*, and *T* are, respectively, the fluid velocity vector, pressure, and temperature; ρ, μ, *k*, and *C* are the density, dynamic viscosity, thermal conductivity, and specific heat capacity, respectively; subscript nf represents a nanofluid property. All fluid properties are calculated at the reference temperature (i.e., the fluid inlet temperature *T_b,i_*).

### 2.2. Physical Properties of the Nanofluid

The physical properties of the nanofluid, including density, heat capacity, thermal conductivity, and viscosity, are defined as follows.

#### 2.2.1. Density and Heat Capacity

Effective density $\rho_{nf}$ of the nanofluid:

(4) $$\rho_{nf}=(1-\phi )\rho_{bf}+\phi \rho_{p}$$

Effective specific heat capacity *C_pnf_* of the nanofluid:

(5) $$C_{pnf}=\frac{(1-\phi ){\left(\rho C_{p}\right)}_{bf}+\phi {\left(\rho C_{p}\right)}_{P}}{\rho_{nf}}$$

In the above two equations, proposed by Pak and Cho [26], $\rho_{bf}$ and $\rho_{p}$ are the mass densities of the base fluid and the nanoparticles, respectively. *C_pbf_* and C*_p__p_* are the specific heats of the base fluid and the nanoparticles, respectively.

#### 2.2.2. Viscosity

The effective viscosity *µ_nf_* of the nanofluid (Masoumi et al. [27]) is:

(6) $$\mu_{nf}=\mu_{bf}+\frac{\rho_{p}V_{B}d_{p}^{2}}{72C\delta }\mu_{bf}$$

where $\mu_{bf}$ is dynamic viscosity of the base fluid; *C* is the correction factor; *V_B_* is the Brownian velocity; *d_p_* is the particle diameter and δ is a volumetric element around a particle. This model was proposed by Masoumi et al. [27] considering the Brownian motion of nanoparticles depending on the temperature, the distance between centers of nanoparticles, and the concentration, as well as the density of nanoparticles.

#### 2.2.3. Thermal Conductivity

The effective thermal conductivity $k_{nf}$ of the nanofluid is [28,29,30,31]:

(7) $$k_{nf}=k_{static}+k_{Brownian}$$

where $k_{static}$ and $k_{Brownian}$ are the thermal conductivity of a static dilute suspension based on Maxwell’s classical correlation [32] and the thermal conductivity due to the Brownian motion, respectively, as shown below:

(8) $$\frac{k_{static}}{k_{bf}}=1+\frac{3(\frac{k_{p}}{k_{bf}}-1)\cdot \phi }{(\frac{k_{p}}{k_{bf}}+2)-(\frac{k_{p}}{k_{bf}}-1)\cdot \phi }$$

(9) $$k_{Brownian}=5\times 10^{4}\beta \phi \rho_{bf}C_{Pbf}g(T,\phi ,d_{p})\sqrt{\frac{k_{B}T}{\rho_{P}d_{p}}}$$

where $k_{bf}$ and $k_{p}$ are the thermal conductivity of the base fluid and the nanoparticle, respectively; $k_{B}$ is a Boltzmann constant; *β* and *g* are two empirical functions [30,31].

The physical properties of water, Al_2_O_3_, and CuO nanoparticles are shown in Table 1. The density ratio, specific heat ratio, viscosity ratio, and thermal conductivity ratio for the nanofluids with various particle volume fractions to base fluid (water) are listed in Table 2.

Al_2_O_3_/water nanofluids with different volume fractions (0.1%, 0.5%, 1%, 1.5%, and 2%) and CuO/water nanofluids with various volume fractions (0.05%, 0.16%, 0.36%, 0.5%, and 0.8%) are used as working fluids. In addition, for comparison, water is also employed as the working fluid. The convective heat transfer coefficient is investigated for various Reynolds numbers in the range of 700 < Re < 1900 in a laminar flow. *Re_nf_*, *Pr_nf_*, and *Pe_nf_* are the Reynolds, Prandtl, and Peclet numbers of the nanofluid, respectively, expressed as:

(10) Renf=ρnfU¯Dhμnf

(11) $${\mathrm{Pr}}_{nf}=\frac{C_{pnf}\mu_{nf}}{k_{nf}}$$

(12) Penf=RenfPrnf=ρnfCpnfU¯Dhknf

where *D_h_* is hydraulic diameter and $\bar{U}$ is average fluid velocity at inlet.

### 2.3. Boundary Conditions

The governing equations of the nanofluid flow are nonlinear coupled partial differential equations. Boundary conditions are specified as follows: at the inlet section, uniform axial velocity $\bar{U}$, temperature *T_in_*, and hydraulic diameter *D_h_* are specified; at the outlet section, the flow and temperature field are assumed to be fully developed. Namely, zero normal gradients prevail for all flow variables except pressure. On the duct wall, the no-slip and constant heat flux boundary conditions are applied.

### 2.4. Solver

ANSYS FLUENT computational fluid dynamics (CFD) software (Cecil Township, PA, USA), incorporated with a finite volume method, is employed to solve the nonlinear governing equations (Equations (1)–(3)) of laminar forced convection heat transfer in a triangular duct with a constant heat flux. The control volume-based technique is used to convert a general scalar transport equation into an algebraic equation that can be solved numerically. It consists of the following procedures: (1) division of the domain into discrete control volumes using a computational grid; (2) integration of the governing equations on the individual control volumes to construct algebraic equations for the discrete dependent variables (“unknowns”) such as velocities, pressure, and temperature; and (3) linearization of the discretized equations and solution of the resultant linear equation system to yield updated values of the dependent variables. Details about the solver algorithms used by ANSYS FLUENT can be found in [33].

**Figure 1:**
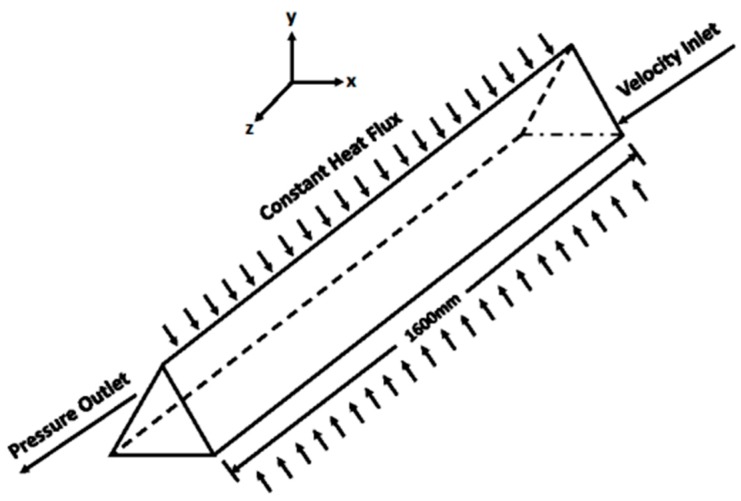
Geometrical configuration in the numerical simulation.

Figure 1 shows the geometrical configuration used in the numerical analysis. A 1.6-m-long duct with a hydraulic diameter of 4.2 mm for Al_2_O_3_/water nanofluids and a 1.6-m-long duct with a hydraulic diameter of 3.5 mm for CuO/water nanofluids are employed, which are exactly the same as those used in Heris et al.’s experiments [4,5]. The Geometry and Mesh Building Intelligent Tool (GAMBIT) [33] model is employed to describe the problem. The model graphs and meshes the spatial domain with a size of 75 × 75 × 800 grids (duct length of 800 and a triangular cross-section area of 75 × 75).

The numerical simulation is carried out at various Reynolds numbers (Peclet numbers) and particle concentrations for Al_2_O_3_ (0.1, 0.5, 1, 1.5, and 2 vol. %) and CuO (0.05, 0.16, 0.36, 0.5, and 0.8 vol. %). The nanoparticle size of Al_2_O_3_ is 20 nm, and that of CuO is 50 nm. The finite volume formulation is used with the Semi-Implicit Method for Pressure-Linked Equations (SIMPLE) algorithm to solve the discretized equations derived from the partial nonlinear differential equations of the mathematical model [33]. The convection terms of the transport equations are discretized by the second-order hybrid central differences/upwind scheme. During the numerical calculation, the residuals of the algebraic discretized equations, resulting from the spatial integration of the conservation equations over finite control volumes, are monitored.

Staggered grid schemes are used in which velocity components are calculated at the points that lie on the center of control volume interfaces and all scalar quantities are calculated at the points that lie in the center of control volume. Pressure and velocity were coupled using SIMPLE. The sequence of operations for the SIMPLE algorithm is as follows:

1. Guess the pressure p*.
2. Solve the momentum equations to obtain u*, v*, w*. Notice that unless the correct pressure field is employed, the resulting velocity field will not satisfy the continuity equation. Such an imperfect field based on a guessed pressure field p* will be denoted by u*, v*, w*.
3. Solve *p′* equation. *p′* is the pressure correction.
4. Calculate corrected pressure *p* (*p* = p* + *p′*).
5. Calculate corrected velocity components *u*, *v*, *w* (*u*= u* + *u′*, *v* = v* + *v′*, w = w* + w*′*). *u′*, *v′*, and *w′* are the velocity corrections for *u*, *v*, and *w*, respectively.
6. Solve other variables (such as *T*).
7. Treat the corrected pressure *p* as a new guessed pressure p*.

Convergence is achieved once the residuals for all discretized equations are smaller than 10^−6^. Then, the average heat transfer coefficient and Nusselt number can be calculated using Equations (13) and (14), respectively:

(13) $$h_{nf}=\frac{C_{pnf}\rho_{nf}\bar{U}A(T_{b,o}-T_{b,i})}{\pi D_{h}L{\left(T_{w}-T_{b}\right)}_{M}}$$

(14) $$Nu_{nf}=\frac{h_{nf}D_{h}}{k_{nf}}$$

where $h_{nf}$ and $Nu_{nf}$ are the average heat transfer coefficient and Nusselt number of the nanofluid, respectively; *L* is the length of the duct; *D_h_* is the hydraulic diameter of the duct; $\bar{U}$ is the mean velocity of the nanofluid at the inlet; (*T_w_* − *T_b_*)*_M_* is the mean temperature difference; *T_b,i_* and *T_b,o_* are the inlet and outlet bulk temperature of the nanofluid, respectively.

## 3. Results and Discussion

### 3.1. Grid-Independence Analysis

**Figure 2:**
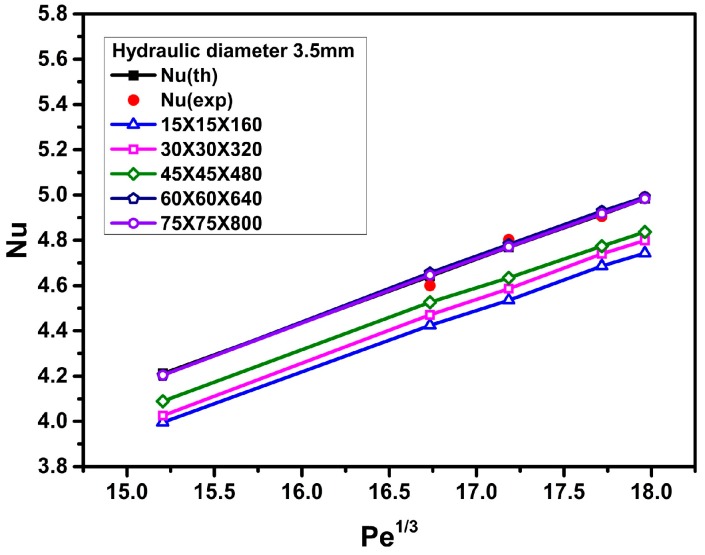
Grid sensitivity testing and comparison among numerical, theoretical, and experimental data for the Nusselt number of water (hydraulic diameter *D_h_* = 4.2 mm).

**Figure 3:**
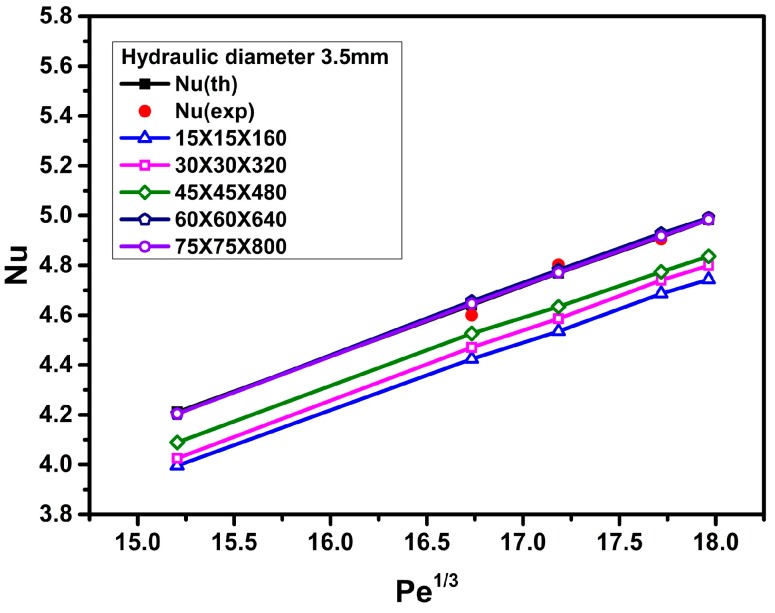
Grid sensitivity testing and comparison among numerical, theoretical, and experimental data for the Nusselt number of water (hydraulic diameter *D_h_* = 3.5 mm).

In order to ensure grid-independent solutions, several non-uniform grids were subjected to an extensive testing procedure. For this purpose, grid densities of 15 × 15 × 160, 30 × 30 × 320, 45 × 45 × 480, 60 × 60 × 640, and 75 × 75 × 800 have been tested and the results of these cases were compared. The effects of the number of mesh points on the Nusselt number of water for a triangular duct with a length of 1.6 m and a hydraulic diameter of 4.2 mm (or 3.5 mm) are shown in Figure 2 (or Figure 3). Based on the results of grid sensitivity testing, the numbers of grid points in the *x*-, *y*-, and *z*-directions are set to 60, 60, and 640, respectively. That is, the numerical results are indeed grid independent since the results reached from the 60 × 60 × 640 grid and the 75 × 75 × 800 grid are nearly identical.

### 3.2. Validation

To validate the accuracy and reliability of the present CFD analysis, the calculated results are compared with the experimental data (Heris et al. [4,5]) and the theoretical results (Equation (15); Sieder-Tate equation [34]) for the Nusselt number versus the Peclet number, using distilled water as the working fluid. Figure 2 and Figure 3 show that good agreements among the computed predictions using a grid density of 60 × 60 × 640, the experimental data [4,5], and the theoretical results, are obtained. The errors are within 1.5% (Figure 2) and 2.1% (Figure 3) for hydraulic diameters of 4.2 mm and 3.5 mm, respectively.

(15) $$Nu=1.86\;{\left({Re}_{nf}{Pr}_{nf}\frac{D_{h}}{L}\right)}^{1/3}{\left(\frac{\mu_{nf}}{\mu_{wnf}}\right)}^{0.14}$$

where $\mu_{wnf}$ is the nanofluid viscosity at the duct wall temperature. The last term in the right-hand side of the above correlation, Equation (15) corrects the coefficient for the effect of a viscosity difference between the bulk fluid and that at the wall (the wall fluid). If the wall is hot, and we consider a liquid is being heated in the duct, the wall temperature and, thus, the temperature of the liquid at the wall, is higher than the bulk temperature. Therefore, the viscosity of the liquid at the wall is less than the bulk viscosity and the boundary layer of the liquid on the wall is thinner, leading to a small increment in the film heat transfer coefficient over that calculated for the constant viscosity case.

### 3.3. Effects of Peclet Number and Particle Volume Concentrations

**Figure 4:**
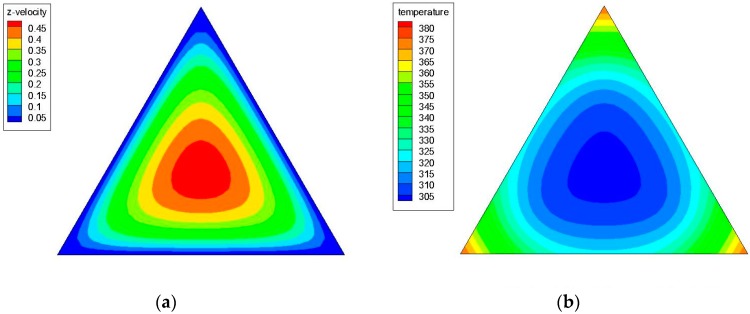
(**a**) Axial velocity and (**b**) temperature contours in a triangular cross-sectional duct with a hydraulic diameter (*D_h_*) of 4.2 mm for water at *z*/*D_h_* = 200 and Re = 1100.

**Figure 5:**
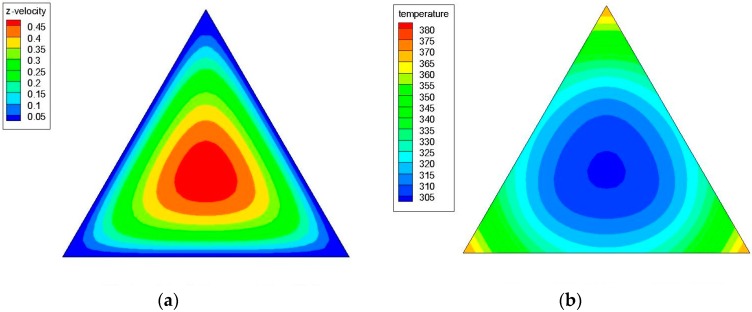
(**a**) Axial velocity and (**b**) temperature contours in a triangular cross-sectional duct with a hydraulic diameter (*D_h_*) of 3.5 mm for water at *z*/*D_h_* = 200 and Re = 1100.

**Figure 6:**
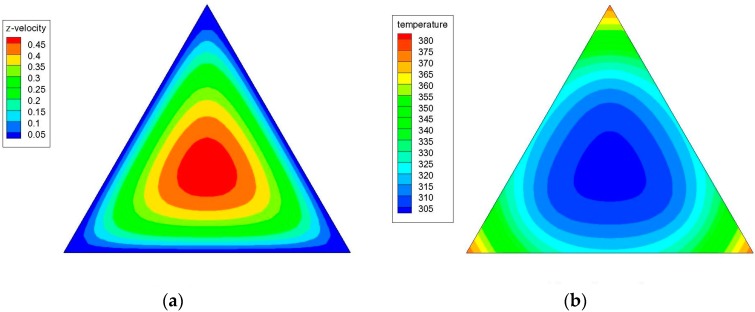
(**a**) Axial velocity and (**b**) temperature contours in a triangular cross-sectional duct with a hydraulic diameter (*D_h_*) of 4.2 mm at *z*/*D_h_* = 200 and Re = 1100 for Al_2_O_3_/water nanofluid with 0.5% nanoparticle volume concentration.

**Figure 7:**
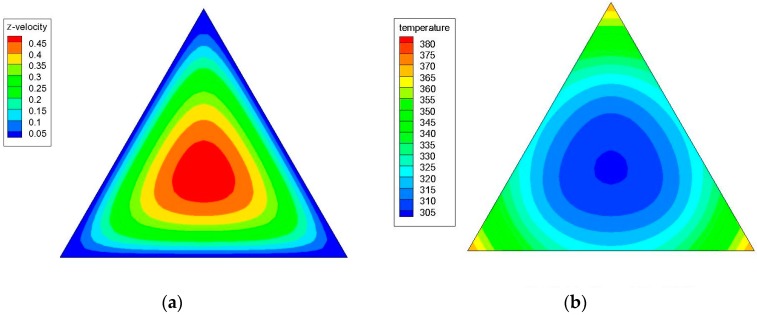
(**a**) Axial velocity and (**b**) temperature contours in a triangular cross-sectional duct with a hydraulic diameter (*D_h_*) of 3.5 mm at *z*/*D_h_* = 200 and Re = 1100 for CuO/water nanofluid with 0.5% nanoparticle volume concentration.

Figure 4a,b shows the axial velocity (the z-component of the velocity) and temperature contours in a triangular cross-sectional duct with a hydraulic diameter (*D_h_*) of 4.2 mm for water at *z*/*D_h_* = 200 and Re = 1100; while Figure 5a,b illustrates the axial velocity and temperature contours in a triangular cross-sectional duct with a hydraulic diameter (*D_h_*) of 3.5 mm for water at *z*/*D_h_* = 200 and Re = 1100. Figure 6a,b shows the axial velocity and temperature contours in a triangular cross-sectional duct with *D_h_* = 4.2 mm at *z*/*D_h_* = 200 and Re = 1100 for Al_2_O_3_/water nanofluid with 0.5% nanoparticle volume concentration; while Figure 7a,b presents the axial velocity and temperature contours in a triangular cross-sectional duct with *D_h_* = 3.5 mm at *z*/*D_h_* = 200 and Re = 1100 for CuO/water nanofluid with 0.5% nanoparticle volume fraction. Notice that the color range from blue to red shows the temperature range from the minimum to the maximum in the triangular duct. As expected, the formation of hot spots that cause less heat transfer performance in sharp corners in triangular ducts can be observed in these figures. Additionally, temperature gradually decreases from the walls to the center of the ducts, but the velocity progressively increases from the walls to the center.

**Figure 8:**
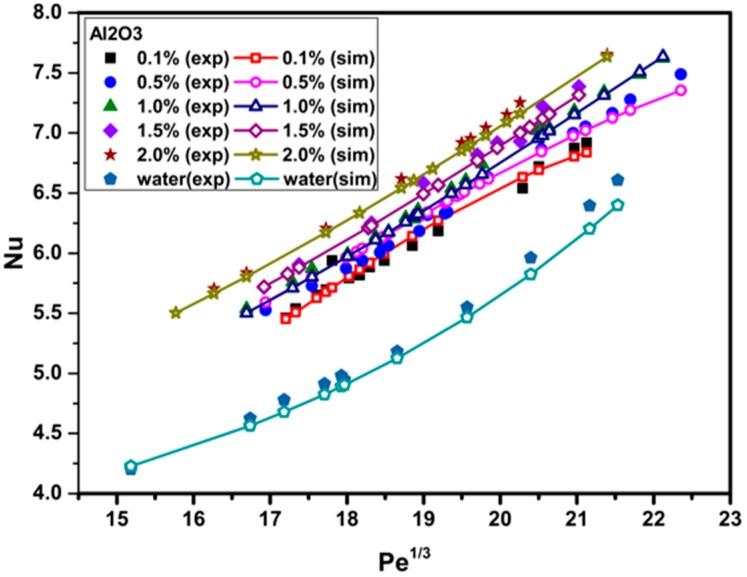
Comparison between numerically-predicted and experimentally-measured Nusselt numbers at various particle volume concentrations and *Pe* values for Al_2_O_3_/water nanofluids.

**Figure 9:**
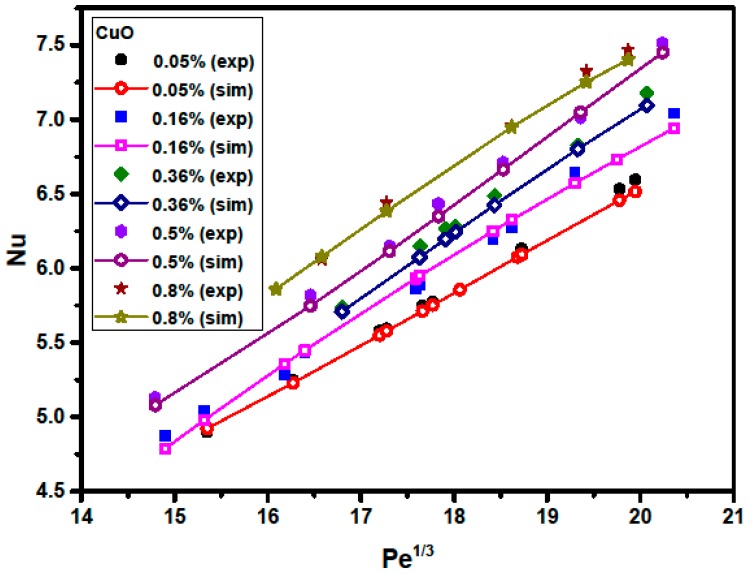
Comparison between numerically-predicted and experimentally-measured Nusselt number at various particle volume concentrations and *Pe* values for CuO/water nanofluids.

Figure 8 and Figure 9 illustrate a comparison between numerical and experimental data for the Nusselt number versus the Peclet number at various particle volume concentrations for Al_2_O_3_/water and CuO/water nanofluids, respectively. It is observed that the Nusselt number significantly increases with increasing particle volume concentration. This is because adding nanoparticles into the base fluid (water) increases the fluid thermal conductivity and the irregular and chaotic movement of the ultra-fine particles increases the energy exchange rates in the fluid [35]. Moreover, increasing the Peclet number leads to an increase in the Nusselt number. A greater Peclet number means higher fluid velocity and a steeper temperature gradient which, in turn, causes the Nusselt number to increase. The heat transfer enhancement of the fluid becomes better at higher Peclet numbers with the use of nanoparticles due to better random motions, collisions, and migration of nanoparticles, especially near the duct corners, through the fluid flow [36,37]. On the other hand, more recent studies indicated that the Nusselt number of the pure base fluid flow and nanofluid flow are well in agreement and can be described without Brownian diffusion [38,39,40,41]. For instance, Utomo et al. [39] reported that, although the nanoparticles affect the thermo-physical properties of the nanofluids, the movement of nanoparticles due to Brownian diffusion and thermophoresis has an insignificant effect on heat transfer coefficient. Martínez-Cuencaco et al. [40] pointed out that the heat transfer enhancement obtained in nanofluids takes place mainly through a Pr number change (viscosity change). Buschmann [41] also concluded that the description of laminar nanofluid pipe flow with inserted twisted tape based on a combination of Reynolds and Prandtl numbers is sufficient because two-phase flow effects, like Brownian and thermophoretic diffusion, are of minor importance.

**Figure 10:**
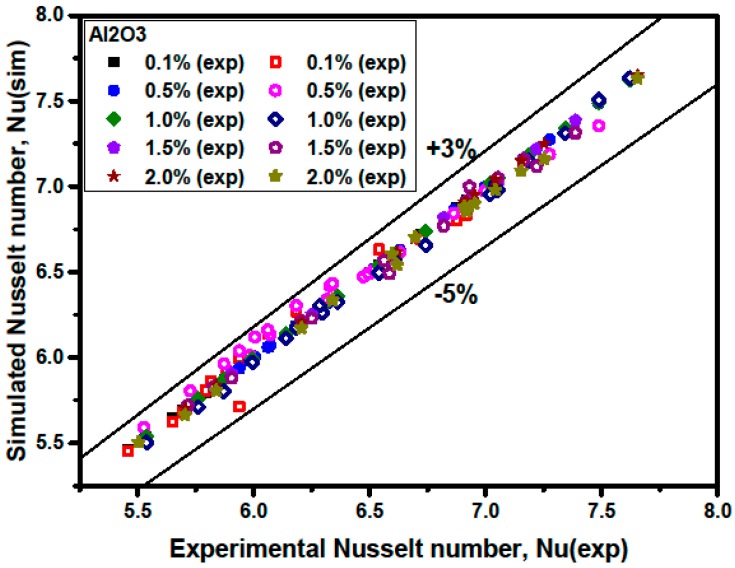
Comparison of measured and predicted Nusselt number for Al_2_O_3_/water nanofluids.

**Figure 11:**
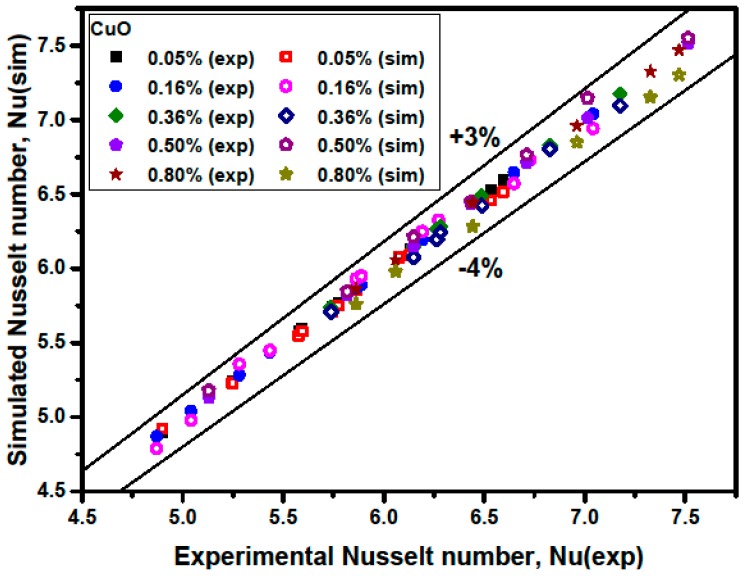
Comparison of measured and predicted Nusselt number for CuO/water nanofluids.

Figure 10 and Figure 11 show a comparison between the simulated average Nusselt numbers Nu(sim) and the experimental Nusselt numbers Nu(exp). The results show that the Nu(sim) coincides well with Nu(exp). Figure 10 and Figure 11 also illustrate that the discrepancies between the simulated average Nusselt numbers and the experimental ones are in the range of −5% to +3% for Al_2_O_3_/water nanofluids, and that the discrepancies are in the range of −4% to +3% for CuO/water nanofluids.

**Figure 12:**
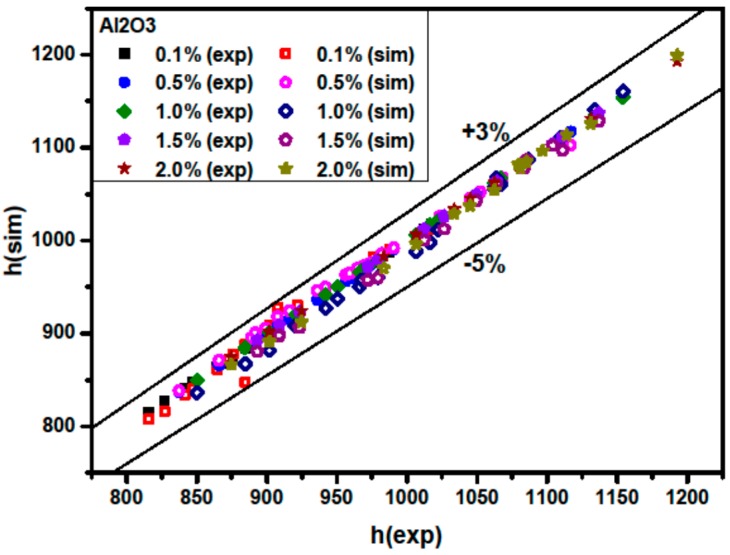
Comparison between numerically-predicted and experimentally-measured heat transfer coefficients at various particle volume concentrations and *Pe* values for Al_2_O_3_/water nanofluids.

**Figure 13:**
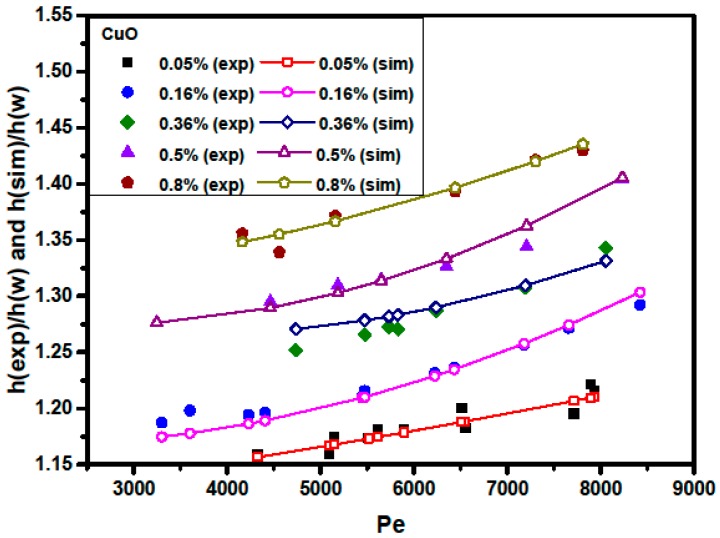
Comparison between numerically-predicted and experimentally-measured heat transfer coefficients at various particle volume concentrations and *Pe* values for CuO/water nanofluids.

Figure 12 (or Figure 13) shows a comparison between the numerical and experimental data for the ratio of the convective heat transfer coefficient of Al_2_O_3_/water (or CuO/water) nanofluid to water versus the Peclet number at various particle volume concentrations. It is clear that the nanofluid with the higher particle volume concentration generates better heat transfer performance (higher heat transfer coefficient), as shown in Figure 12 and Figure 13. For instance, as can be seen in Figure 12, at a Peclet number of 5000, increasing the particle volume fraction from 0.1% to 2%, the ratio of the convective heat transfer coefficient of Al_2_O_3_/water nanofluid to water increases from 1.151 to 1.342, corresponding to a 16.6% growth of heat transfer enhancement.

Additionally, heat transfer enhancement is increased as the Peclet number increases. For example, as shown in Figure 13, at a particle volume fraction of 0.5%, increasing the Peclet number from 5000 to 7000, the ratio of the convective heat transfer coefficient of CuO/water nanofluid to water increases from 1.299 to 1.355, corresponding to a 4.3% growth of heat transfer enhancement. The increment of Pe with the nanofluid flow rate causes the convective heat transfer enhancement to increase, which may result from better chaotic motion and nanoparticle migration, especially near the duct corners [36,37]. Figure 12 also shows that at Pe = 7000, a 35% enhancement in the convective heat transfer coefficient can be obtained for an Al_2_O_3_/water nanofluid with 2% particle volume concentration when compared to pure water. Moreover, as can be seen in Figure 13, at the same Peclet number (Pe = 7000), a 41% enhancement in the convective heat transfer coefficient is achieved for a CuO/water nanofluid with 0.8% volume concentration, as compared with pure water.

Notice that at Pe = 7000 and ϕ = 2.0 vol. %, the augmentation of the heat transfer coefficient of water-based Al_2_O_3_ nanofluids (35%) is much larger than that of effective thermal conductivity (6.37%, as shown in Table 2), predicted by Equation (7) [28,29,30,31]. Similarly, at Pe = 7000, the heat transfer coefficient of water-based CuO nanofluids is increased by 41% at ϕ = 0.8 vol. % compared to pure water, and the enhancement of the heat transfer coefficient is much higher than that of the effective thermal conductivity (7.15%, as shown in Table 2) at the same volume concentration, calculated by Equation (7). Therefore, in addition to increased thermal conductivity, other mechanisms, such as viscosity change, thinner thermal boundary layer, random movement and migration of nanoparticles, and energy transfer by nanoparticle dispersion, may be responsible for the heat transfer enhancement of nanofluids.

**Figure 14:**
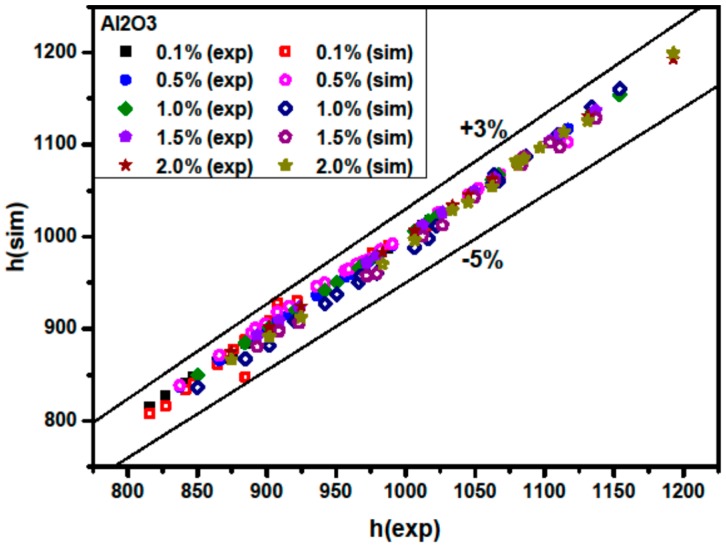
Comparison of the measured and predicted heat transfer coefficient to the water heat transfer coefficient for Al_2_O_3_/water nanofluids.

Figure 14 shows a comparison between h(sim) and h(exp) for Al_2_O_3_/water nanofluids. Here h(exp) and h(sim) are experimental and simulated average nanofluid heat transfer coefficients, respectively. It is found that the simulated average nanofluid heat transfer coefficient coincides well with the experimental average nanofluid convective heat transfer coefficient [4]. The discrepancies are in the range of −5% to +3%. It is also seen that the heat transfer coefficient increases with the Peclet number and with higher particle volume concentration. Explanations of heat transfer enhancement at higher Peclet numbers and higher particle volume concentrations are discussed above.

**Figure 15:**
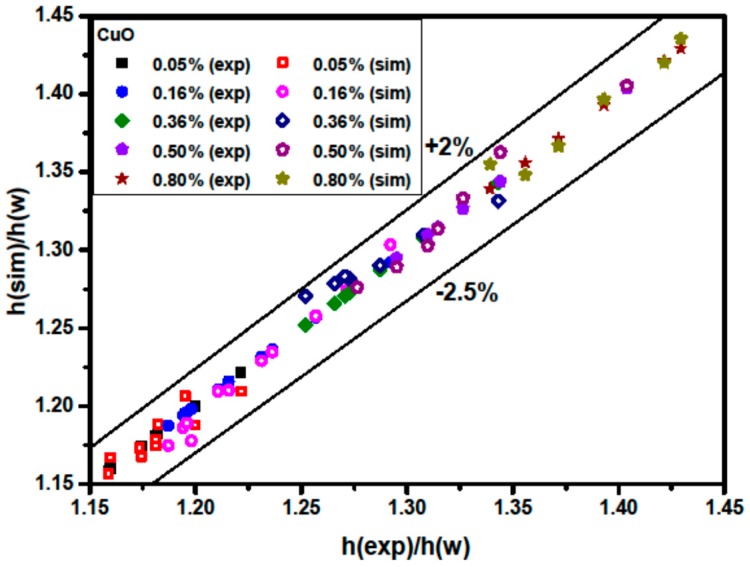
Comparison of the measured and predicted heat transfer coefficient to the water heat transfer coefficient for CuO/water nanofluids.

Figure 15 illustrates a comparison between h(sim)/h(w) and h(exp)/h(w) for Al_2_O_3_/water nanofluids, where h(w) is the theoretical average water convective heat transfer coefficient calculated from the Sieder-Tate equation [24]. h(sim)/h(w) denotes the ratio of the simulated average nanofluid heat transfer coefficient to the theoretical average water heat transfer coefficient calculated from Sieder-Tate equation, and the h(exp)/h(w) designates the ratio of the experimental average nanofluid heat transfer coefficient to the theoretical average water heat transfer coefficient calculated from the Sieder-Tate equation. It is found that the ratio of h(sim)/h(w) is in good agreement with that of h(exp)/h(w). The discrepancies are in the range of −2.5% to +2%.

## 4. Conclusions

Laminar forced convection of Al_2_O_3_/water and CuO/water nanofluids in an equilateral triangular cross-sectional duct subjected to constant heat flux is numerically studied. The results show that heat transfer coefficient and Nusselt number increase with increasing Peclet number and particle volume concentration. At Pe = 7000, a 35% enhancement in the convective heat transfer coefficient can be obtained for an Al_2_O_3_/water nanofluid with 2% particle volume concentration when compared to pure water. At the same Peclet number, a 41% enhancement in the convective heat transfer coefficient can be achieved for a CuO/water nanofluid with 0.8% particle volume concentration, as compared with pure water. The augmentation of the heat transfer coefficient of both Al_2_O_3_/water and CuO/water nanofluids is much higher than that of effective thermal conductivity. Therefore, in addition to the increased thermal conductivity, other mechanisms (such as viscosity change, thinner thermal boundary layer, random movement and migration of nanoparticles, and energy transfer by nanoparticle dispersion) may be responsible for the heat transfer enhancement of nanofluids. Furthermore, theoretical correlations calculated from the Sieder-Tate equation are not able to predict nanofluid thermal performance. Moreover, the numerical results are found to be in good agreement with published experimental data [4,5]. The discrepancies between the simulated average Nusselt numbers and the experimental ones are in the range of −5% to +3% for Al_2_O_3_/water nanofluids and in the range of −4% to +3% for CuO/water nanofluids.

## Figures and Tables

**Table 1:** Thermophysical properties of base fluid and nanoparticles at 298 K.

Property	Basic Fluid (Water)	γ-Al_2_O_3_	CuO
Specific heat (J/kg K)	4182	880	535.6
Density (kg/m^3^)	998.2	3890	6350
Thermal conductivity (W/m K)	0.597	46	69
Viscosity (kg/ms)	9.93 × 10^−4^	-	-

**Table 2:** The density ratio, specific heat ratio, viscosity ratio, and thermal conductivity ratio for the nanofluids with various particle volume fractions to base fluid (water) at 298 K.

Nanoparticle	ϕ (%)	$\rho_{nf}/\rho_{bf}$	$C_{pnf}/C_{pbf}$	$\mu_{nf}/\mu_{bf}$	$k_{nf}/k_{bf}$
Al_2_O_3_	0.1	1.0029	0.9968	1.0843	1.0031
	0.5	1.0145	0.9844	1.1557	1.0157
	1.0	1.0289	0.9692	1.2178	1.0315
	1.5	1.0434	0.9544	1.2802	1.0475
	2.0	1.0579	0.9400	1.3521	1.0637
CuO	0.05	1.0027	0.9972	1.0184	1.0045
	0.16	1.0086	0.9912	1.0275	1.0143
	0.36	1.0193	0.9804	1.0371	1.0322
	0.50	1.0269	0.9729	1.0423	1.0447
	0.80	1.0430	0.9574	1.0519	1.0715

## Nomenclature

A	Surface area of square cross-section duct (m^2^)
*C*	Specific heat (kJ·kg^−1^·K^−1^)
*D_h_*	Hydraulic diameter (m)
h(exp)	Experimental average nanofluid heat transfer coefficient (W·m^−2^·K^−1^)
h(sim)	Simulated average nanofluid heat transfer coefficient (W·m^−2^·K^−1^)
*k*	Thermal conductivity (W·m^−1^·K^−1^)
*L*	Duct length (m)
Nu(exp)	Average nanofluid Nusselt number obtained from experiments
Nu(sim)	Average nanofluid Nusselt number calculated from CFD analysis
*Pe*	Peclet number
*Pr*	Prandtl number
*q*	Heat flux (W/m^2^)
*Re*	Reynolds number
*T_b_*	Bulk temperature (K)
*T_w_*	Duct wall temperature (K)
$\bar{U}$	Average fluid velocity (m·s^−1^)
*u*	The x-component of the velocity (m^−1^·s^−1^)
*v*	The y-component of the velocity (m^−1^·s^−1^)
*w*	The z-component of the velocity (m^−1^·s^−1^)
Greek Symbols	
γ	Ratio of the nano-layer thickness to original particle radius
*μ*	Viscosity (Pa s)
*μ*_wnf_	Nanofluid viscosity at duct wall temperature (Pa s)
ϕ	Nanoparticle volume fraction (%)
ρ	Density (kg·m^−3^)
Subscripts	
bf	Base fluid
i	Inlet
nf	Nanofluid
o	Outlet
p	Solid nanoparticles
w	Wall
